# The portrait of Familial Mediterranean Fever in N. Greek pediatric patients: a 30-year experience

**DOI:** 10.1186/1546-0096-13-S1-P106

**Published:** 2015-09-28

**Authors:** P Pratsidou-Gertsi, M Trachana, V Sgouropoulou, E Farmaki, V Tzimouli, G Pardalos, F Kanakoudi-Tsakalidou

**Affiliations:** 1 Pediatric Immunology and Rheumatology Referral Center, Ippokration Hospital, First Department of Pediatrics, Aristotle University, Thessaloniki, Greece

## Introduction

Familial Mediterranean Fever (FMF), is the second commonest autoinflammatory disease in pediatric Greek patients (pts) after PFAPA. So far, long-term follow-up case series in Greek FMF pts have not been emerged.

## Objectives

To depict: a) the clinical phenotype, the genotype-phenotype interplay and b) the response to treatment and the long-term outcome in a large series of Greek FMF pts.

Patients and methods: Seventy children (46 females) with established FMF according to Tel Hashomer classification criteria were enrolled in this retrospective study. Pts were followed-up over a 30-year period (1986-2015) and most of them underwent a genetic molecular analysis using either FMF strip assay (Vienna Lab) or NIRCA or recently NGS.

## Results

The mean age at FMF onset was 3.54 years (range 0.83-18.5), the mean lag time 36.72 months (range 91-185) and the cumulative follow-up time 731.53 years (9.88/pt). A positive family history was recorded in 27/70 pts (38.6%).

In respect to phenotype, a typical phenotype was recorded in 75.7%; the commonest manifestations at onset were periodic fevers (100%), abdominal pain (84.3%), rheumatic attacks (arthritis or arthralgias in 52.85%) and chest pain (40%), while monosymptomatic were only 5.7%. Genotyping studies traced ≥ 1mutation in 63/67 pts; compound heterozygotes or homozygotes were 22 and 13 pts respectively. The commonest mutations were M694V (51.6%) and M680I (38.7%), the combination M694V/M680I, the most frequent one (19%) and M694V was significantly correlated with rheumatic manifestations (p=0.003). A complete or partial response to colchicine was recorded in 51.5% and 42.4% respectively, whereas unresponsiveness was observed in 6%. One refractory patient responded to anti-IL1β administration and no patient ever developed amyloidosis.

## Conclusions

In this case series, the FMF phenotype in N. Greece was a typical one and not differential from other Caucasian ethnicities, although it was milder and absent of amyloidosis. The genotype distribution was in line with previously described mutations in other Mediterranean Countries. The systemic follow-up and contemporary management in an organized Center hinders non-compliance and contributes to an optimal disease outcome.

